# Fiberoptic-guided nerve integrity monitoring tube intubation assisted by video-laryngoscope with external laryngeal manipulation in a patient with anteriorly displaced larynx due to huge goiter with retropharyngeal involvement: A case report

**DOI:** 10.1097/MD.0000000000029041

**Published:** 2022-03-11

**Authors:** Ji-Yoon Kim, Ji-Yong Yeom, Si-Jeong Youn, Jeong-Eun Lee, Jin-Young Oh, Sung-Hye Byun

**Affiliations:** a Department of Anesthesiology and Pain Medicine, College of Medicine, Chung-Ang University, Seoul, Republic of Korea; b Department of Anesthesiology and Pain Medicine, School of Medicine, Kyungpook National University, Daegu, Republic of Korea.

**Keywords:** difficult airway, external laryngeal manipulation, fiberoptic bronchoscopy, goiter, videolaryngoscopy

## Abstract

**Rationale:**

Goiter, an abnormal enlargement of the thyroid gland, can induce airway distortion or tracheal compression. Airway management can be challenging for anesthesiologists, depending on the location and size of the mass as well as the patient's airway conditions, although it is reported that most cases can easily be managed by oral intubation.

**Patient concerns:**

A 61-year-old female patient who had planned for a total thyroidectomy due to a huge goiter was intubated with nerve integrity monitoring (NIM) tubes, using video laryngoscopy (VL) and oral fiberoptic bronchoscopy (FOB) alone. The respective attempts initially failed.

**Diagnosis:**

The patient's thyroid mass extended from the C3 cervical spine level to the T1 thoracic spine level with retropharyngeal involvement, causing an upper airway anatomical alteration that made intubation difficult. FOB manipulation was challenging due to the acute angulation of the laryngeal inlet and the tongue and the consequent interruption by the epiglottis. There was resistance to tube introduction, despite counterclockwise rotation of the NIM tube, due to acute angulation of the larynx and circumferential narrowing of the oropharyngeal and supraglottic space.

**Interventions:**

In the first step of FOB-guided intubation, external laryngeal manipulation (ELM) was performed to improve the angle of the glottic opening and to elevate epiglottis tip. This allowed for FOB introduction into the trachea. VL was then performed transorally to elevate the tongue base and increase space, using the blade. ELM was applied simultaneously to move the glottis lower, thereby reducing the angle of the tube passage.

**Outcomes:**

The NIM tube was successfully introduced into the trachea with counterclockwise rotation in FOB-guided intubation.

**Lessons:**

The combination of techniques using basic and popular devices and maneuvers, such as ELM and VL, may be useful for the successful management of difficult airways related to retropharyngeal goiter, without the need for surgical airway.

## 1 Introduction

Goiter refers to an abnormal enlargement of the thyroid gland, and the mass can induce airway distortion or tracheal compression. Fortunately, most cases of goiter can easily be managed by oral intubation.^[1]^ Nevertheless, airway management can be challenging for anesthesiologists, depending on the size and location of the mass.^[2,3]^ General guidelines for management of difficult airways are well established,^[4]^ and airway difficulty encountered in patients with goiter has been resolved using various approaches.^[2,3,5]^

Herein, we present a case of a patient with acute angulation of the larynx and circumferential narrowing of the oropharyngeal and supraglottic space, due to a huge retropharyngeal goiter. Initial attempts of nerve integrity monitoring (NIM) tube intubation using videolaryngoscopy (VL) and oral fiberoptic bronchoscopy (FOB) alone had failed. We mounted the NIM tube within the patient's trachea successfully by combining external laryngeal manipulation (ELM) and VL to FOB.

## 2 Case presentation

**Figure 1:**
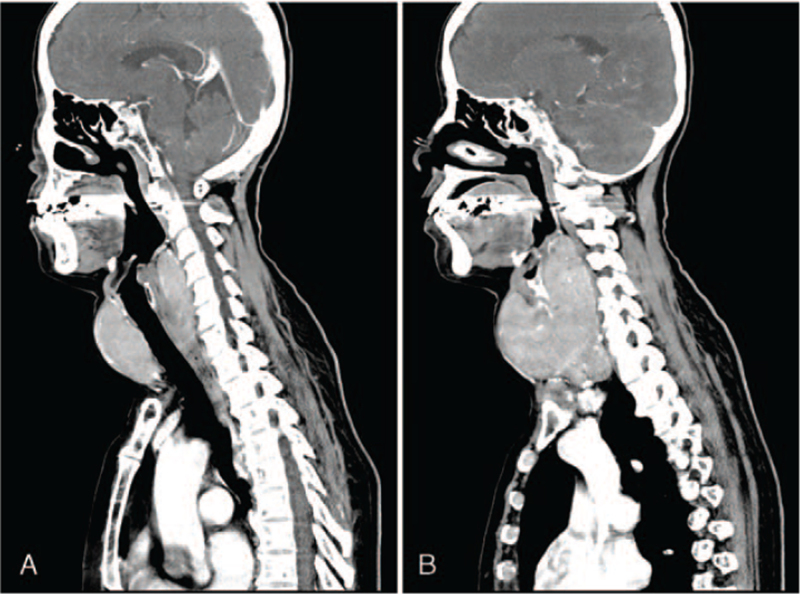
The thyroid goiter that extended from the C3 level to the T1 level and occupied the retropharyngeal space, resulting in anterior displacement of the larynx is shown in the sagittal view of neck computed tomography. An image of the midline plane is shown in (A), and the image of the plane that was shifted slightly to the right from the midline is shown in (B).

**Figure 2:**
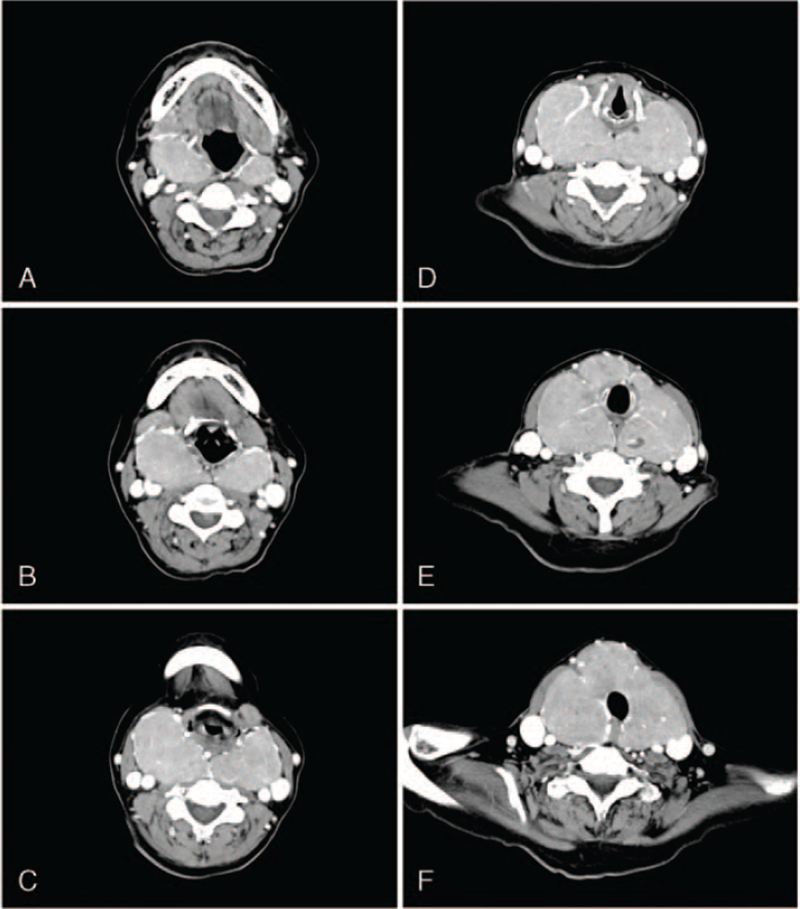
The huge goiter with bilateral retropharyngeal involvement and the trachea that was deviated leftward slightly without narrowing are seen in a series of axial computed tomography scans of the neck, showing the region from the upper structures (A) to the lower structures (F). The narrowest part of the patient airway was measured as 6.85 mm of the anteroposterior diameter and 13.45 mm of the transverse diameter in (C).

The case presents a 61-year-old female patient (height: 150 cm, weight: 42.4 kg, body mass index: 18.4 kg/m^2^) with a severe diffuse goiter intended for total thyroidectomy. The patient was diagnosed with hyperthyroidism 20 years prior to treatment at our facility and had been previously treated with methimazole, an antithyroid medication. The goiter had 3 years of growth, and the patient's thyroid function had not been well-controlled; therefore, surgical treatment was recommended. The patient's preoperative thyroid-stimulating hormone level was suppressed to 0.01, and free thyroxine was normal at 0.95. Neck computed tomography revealed that the thyroid mass extended superiorly to the C3 cervical spine level and inferiorly to the T1 thoracic spine level. The retropharyngeal space was involved as well, causing anterior displacement of the upper airway (Fig. 1). The trachea was deviated slightly to the left due to the mass, but there was no narrowing within the trachea (Fig. 2A-F). On preoperative airway evaluation, the patient's Mallampati classification (MP) was 3, thyromental distance was 5.5 cm, the inter-incisor gap was 5 cm, the range of neck extension was fair, and the condition of teeth was acceptable with no teeth malocclusion. There were no signs of airway narrowing, including abnormal breathing sound or breathing difficulty, even while lying down.

Upon arrival to the operating room, standard monitoring, including noninvasive blood pressure, electrocardiogram, and peripheral oxygen saturation, was performed. A bispectral index (BIS) senser was placed on the patient's forehead and used to monitor the depth of anesthesia. Using 100% O_2_ via a face mask, preoxygenation was performed. After intravenous injection of lidocaine 40 mg, anesthesia was induced with a propofol effect-site concentration (Ce) of 4.0 mcg/mL and remifentanil Ce of 4.0 ng/mL as a target. A NIM tube (NIM EMG Endotracheal Tube, Medtronic Xomed Inc., Jacksonville, FL) was prepared for intraoperative neuromonitoring in order to protect recurrent laryngeal nerve. Furthermore, to verify that NIM tube electrodes were appropriately placed and an appropriate EMG signal was detected before surgery, the surgeon requested that a small dose of neuromuscular blocking agent (NMBA) be used. ED_95_ of Rocuronium, measuring 0.3 mg/kg, was administered when BIS dropped below 60, and sufficient time was given for NMBA in the anticipation of delayed Rocuronium onset. Although the quality of manual ventilation was not excellent, mechanical ventilation was well performed with the face mask fitted while providing jaw thrust.

After 4 to 5 minutes of NMBA administration and when it was verified that the train-of-four count was 3 (propofol Ce 5.0 mcg/mL, BIS 40 at the time), transoral intubation was performed using a McGrath MAC (Aircraft Medical Ltd., Edinburgh, UK) video laryngoscope with blade size 3 while patient's head was placed on a pillow making sniffing position. According to the VL view, the glottic opening was located in the upper part of the screen, and the posterior extremity of the glottis was barely seen. Oropharyngeal and supraglottic spaces seemed insufficient in using a bent NIM tube with an internal diameter (ID) of 7.0 mm, due to the circumferential thyroid mass, and the stylet angle was inappropriate to insert the tube toward the glottis. In the second attempt, we changed the size of the NIM tube to an ID of 6.0 mm, and applied McGrath MAC X blade size 3, which had a more acute curvature and a slimmer width compared with McGrath MAC blade size 3. The laryngeal view was improved (the glottis was entirely visible), but the route through which the tube had to be inserted was still at an acute angle and narrow; thus, it was difficult for the bent NIM tube with preloaded stylet to proceed toward the glottis. In our third attempt, we decided to use FOB, but FOB manipulation during a jaw thrust maneuver to maintain airway patency, which was performed by the assistant anesthesiologist, was challenging owing to the acute angulation of the laryngeal inlet and tongue and consequent interruption of the FOB view by the epiglottis. Therefore, another assistant anesthesiologist performed ELM to improve the angle of the glottic opening and to elevate the epiglottis tip, which allowed for FOB introduction into the trachea. However, there was a resistance to tube introduction during the next step of tube railroading over FOB, despite counterclockwise rotation of the NIM tube. It was believed that this problem was due to the acute-angled larynx as well as circumferential narrowing of the oropharyngeal and supraglottic space. To overcome this problem, an assistant anesthesiologist inserted a McGrath MAC X blade size 3, which was used in previous attempts, into the patient's mouth using the left hand. While elevating the tongue base using the blade to make more space available, ELM was used simultaneously with the right hand to move the glottis lower and to reduce the angle of the tube passage. Along with these maneuvers, the attending anesthesiologist rotated the NIM tube counterclockwise and was able to introduce it into the trachea after 2 attempts.

Anesthesia was maintained by total intravenous anesthesia using propofol and remifentanil during surgery, and dexamethasone 10 mg was injected to prevent airway edema from multiple attempts of intubation. Total thyroidectomy was performed successfully without any intraoperative events. The surgically excised mass was 10.0 × 5.0 × 3.6 cm for the right lobe and 8.0 × 7.0 × 4.0 cm for the left lobe, with a weight of 180 g and 110 g, respectively. We did not expect any difficulty with the airway management when waking up the patient, as the goiter was surgically removed, and the patient was awakened safely with a remifentanil Ce of 1.5 ng/mL maintained for smooth emergence from anesthesia. Two hours after the surgery, the patient was re-admitted to the operating room for neck swelling due to postoperative bleeding. Although the neck was very swollen, O_2_ was well maintained at SpO_2_ 98% to 100% with O_2_ measuring 10 L/min. There was no issue with ventilation, and the surgeon disinfected the surgery area to remove sutures and the hematoma before induction. Anesthesia was induced with thiopental 4 mg/kg, and succinylcholine 1 mg/kg was injected. Intubation was successfully done using McGrath MAC blade size 3 and an armored endotracheal tube of ID 6.0 mm. The glottis was very well observed via the VL monitor. Bleeding focus was the left inferior thyroid artery branch, which was ligated, and the surgery was completed without additional events. The patient has provided informed consent for the publication of this case report.

## 3 Discussion

Retropharyngeal goiter, which occupies the retropharyngeal space, can induce circumferential narrowing of the epiglottis and supraglottic larynx compression^[3]^ and contribute to the interference of the laryngeal inlet, unlike substernal goiter, which extends to the mediastinum, where the trachea is primarily impacted. The anteriorly-displaced laryngeal inlet can be exhibited due to the mass involving retropharyngeal space, which can aggravate upper airway compression caused by impingement of supraglottis. In patients with these anteriorly-displaced glottic openings, visualization of the glottis with direct laryngoscopy (DL) using the conventional laryngoscope with a Macintosh or Miller blade may be difficult or even impossible. The ability to visualize the glottis can affect the ease of tracheal intubation, and it is known that using VL allows for more glottic exposure than DL and increases the intubation success rate; therefore, VL is, recommended in cases of difficult airway management.^[4,6]^ However, in some cases, the glottis may not be well-visible even with VL as shown in the present case, or the actual introduction of an endotracheal tube may be challenging in practice despite obtaining a good glottic view on a VL monitor. Even in the FOB-guided intubation procedure, which we performed as an alternative to VL, FOB and endotracheal tube (ETT) placement into the trachea was difficult. Therefore, additional techniques may be needed to overcome these challenges.

Jaw thrust maneuver allows displacement of the mandible forward and lifts up the hyoid bone, which then lifts the tongue root connected to the hyoepiglottic ligament and the epiglottis from the posterior pharyngeal wall. This provides more space in the oropharyngeal cavity,^[6–8]^ and it expands and opens the laryngeal inlet itself.^[6,8,9]^ Therefore, jaw thrust has beneficial effects in improving glottic visualization in both the VL view^[10]^ and the FOB view.^[11]^ When using this maneuver, FOB insertion into the trachea as the first step of FOB-guided intubation and ETT advancement into the trachea as the second step can be easily performed.^[6]^ Consequentially, jaw thrust is reported to increase the success rate of intubation.^[11]^ However, there are also opinions that this maneuver may make it more difficult for the ETT to pass into the trachea, and the although the reason for this is unclear, it may be because jaw thrusting shifts the larynx anteriorly, which widens the esophageal inlet.^[9]^ Another technique, ELM, is a representative maneuver for improved laryngeal view,^[12,13]^ the effectiveness of which has been demonstrated in many studies.^[14–16]^ ELM is also reported as the most commonly used technique amongst anesthesiologists for managing difficult laryngoscopy.^[17]^ ELM improves the line of sight alignment by exerting posterior pressure to the thyroid cartilage, to which the vocal cord is attached, and by reducing the anterior tilt of the larynx.^[15,16]^ At the same time, ELM exerts pressure indirectly to the hyoepiglottic ligament, which causes the ligament to push the epiglottis forward, allowing exposure of the glottis opening.^[13,15,16]^ ELM is a separate procedure from Sellick's maneuver, which is exerted on cricoid cartilage to prevent passive regurgitation of gastric contents and pulmonary aspiration.^[18]^ When the cricoid cartilage below thyroid cartilage is pressed, this can increase the anterior tilt of the larynx. Therefore, caution should be practiced as this maneuver may worsen the laryngeal view.^[19]^ In our patient, FOB insertion, which is the first step in FOB-guided intubation, was difficult to perform with jaw thrust only, due to the anteriorly placed, acute-angled larynx. By adding ELM, the airway angulation was minimized and the epiglottis was opened by pushing upwards, which allowed for success in the first step of FOB-guided intubation.

However, the success of this first step does not guarantee successful tracheal intubation, and while there are multiple factors that interfere with the tube advancing over FOB, the second step, bevelled tip of ETT, can impinge on the vocal cords, arytenoid cartilages, or other hypopharyngeal structures and cause difficulties in the tube passage (the right arytenoid cartilage may be considered as the site that is most frequently impinged, especially during oral FOB-guided intubation).^[9,20]^ To overcome these difficulties, tube rotation, changes in the patient's head or neck position, jaw thrust maneuver, ELM, and lingual traction are helpful.^[21]^ In particular, tube rotation in the 90 degree counterclockwise direction brings bevelled ETT and FOB in close contact, thus minimizing impingement.^[9]^ Previous studies report that impingement can be overcome with 90 degree counterclockwise rotation of tube regardless of the impingement site.^[22]^ In our patient, the supraglottic space was narrowed from the enlarged thyroid mass that expanded the potential impingement site from the hypopharynx to the laryngeal inlet. To insert a relatively thin FOB into the trachea, jaw thrusting was helpful by widening the laryngeal inlet space. However, the maneuver did not allow for sufficient space and angle to introduce a relatively thick NIM tube in rotation.

Several studies report improved level of difficulty when laryngoscope is combined during FOB-guided intubation,^[23]^ and by using laryngoscope, effects of both jaw thrust and lingual traction can be expected and the airway can be cleared.^[23]^ There are a few case reports discussing the management of difficult airways, such as cervical spine ankylosis due to rheumatoid arthritis or large vallecular hemangiomas, by using DL- or VL-assisted FOB-guided intubation.^[24,25]^ Also, there are case reports of using DL and ELM together, to assist FOB-guided intubation and to overcome airway obstruction from perilaryngeal edema.^[26]^ In our patient, a McGrath video laryngoscope was inserted, and the jaw and the tongue were lifted with the blade to expand the space for the rotating NIM tube to pass through the airway. To improve larynx angulation, the ELM procedure that was previously used was also implemented. VL has some advantages over DL, including the fact that the FOB tip can be displayed, allowing for confirmation of the tube entry problems and simultaneous feedback, whereby the assistant anesthesiologist can implement appropriate location and pressure.

Appropriate airway management in patients with retropharyngeal thyroid goiter has not been defined.^[3]^ There are cases where DL, VL, and FOB failed, depending on the patient's airway conditions, and surgical airway interventions were needed.^[2]^ Other cases have required surgery with awake tracheostomy.^[3]^ However, the best approach for securing the airway in such cases usually includes standard DL intubation or FOB-guided intubation. Previous publications regarding airway management in patients with goiter recommended planning for difficult airways management by considering the location of the goiter or the presence of factors that can increase the incidence of difficult intubation.^[5]^ Kaur et al^[27]^ mentioned that the factors that predicted very difficult mask ventilation and intubation in their patient with goiter included obesity, retrognathia, decreased mouth opening, protruding teeth, large tongue, MP 4, decreased neck movement, and dyspnea while lying down indicating tracheal compression and awake FOB-guided intubation (AFOI) rather than DL was planned for the patient. However, according to the studies of Loftus et al^[28]^ and Gilfillan et al,^[29]^ AFOI performed as an initial attempt failed in 12%∼16% of patients with goiter. Rather, such patients could be successfully intubated using DL or VL after intravenous induction,^[28]^ and it was described that AFOI requires patient cooperation and that topical airway anesthesia can aggravate respiratory depression.^[29]^ If difficult intubation is strongly expected due to the presence of symptoms of airway narrowing or several predicting factors on preoperative evaluation, AFOI with sufficient topical anesthesia or awake tracheostomy by ENT surgeons should be considered. However, when using the NIM tube, it is recommended to avoid the use of lubricants, such as topical lidocaine,^[30]^ although AFOI should not be excluded in very dangerous airways even in situations where NIM tubes are used, and such experience of AFOI using NIM tube have already been presented and published as a report.^[31]^ In addition, the NIM tube's electrode must make contact with the vocal cord for the intraoperative neuromonitoring of the recurrent laryngeal nerve, and in patients who have undergone tracheostomy, this can be cumbersome as a retrograde tracheal intubation technique must be used to mount such a tube. On preoperative airway evaluation of our patient, there were few factors predicting intubation difficulty except MP 3 and thyromental distance <6 cm, and there were no symptoms suggesting airway narrowing. Although the narrowest part of the airway on the neck computed tomography was measured with an anteroposterior diameter of 6.85 mm, it was thought to be the narrowed entrance which was partially hidden with the drooping epiglottis, and the transverse diameter of 13.45 mm was expected to allow the NIM tube passage (Fig. 2C). Moreover, the patient looked anxious, albeit not severe, and considering such patient's overall situations, we planned to perform intubation using VL instead of AFOI or awake tracheostomy. As a rescue intubation technique, considering the patient's situation, the aforementioned less invasive airway devices and maneuvers were combined and applied without introducing a surgical airway to successfully secure an airway.

In conclusion, in our patient with an anteriorly displaced larynx and circumferentially narrowed supraglottic space due to a huge goiter with retropharyngeal involvement, NIM tube was successfully intubated with oral FOB-guided intubation, a basic method, with the assistance of a simple ELM procedure and a popularized VL. The combination of techniques using these basic and popular devices and maneuvers could be useful in managing difficult airways related to retropharyngeal goiter without the need for surgical airways.

## Author contributions

**Conceptualization:** Ji-Yoon Kim, Si-Jeong Youn, Sung-Hye Byun.

**Data curation:** Ji-Yong Yeom, Sung-Hye Byun.

**Investigation:** Ji-Yoon Kim, Ji-Yong Yeom, Si-Jeong Youn, Jeong-Eun Lee, Sung-Hye Byun.

**Supervision:** Jeong-Eun Lee, Jin-Young Oh, Sung-Hye Byun.

**Visualization:** Sung-Hye Byun.

**Writing – original draft:** Ji-Yoon Kim, Sung-Hye Byun.

**Writing – review & editing:** Ji-Yoon Kim, Sung-Hye Byun.

## References

[1] Sajid B Rekha K. Airway management in patients with tracheal compression undergoing thyroidectomy: a retrospective analysis. Anesth Essays Res 2017;11:110–6. 28298767 10.4103/0259-1162.186608 PMC5341636

[2] Baik FM Zhu V Patel A Urken ML. Airway management for symptomatic benign thyroid goiters with retropharyngeal involvement: need for a surgical airway with report of 2 cases. Otolaryngol Case Rep 2018;7:10–2.

[3] Thomas CM Mattingly JK Hendrickse A Song JI. Case report of a massive retropharyngeal goiter resulting in laryngeal compression. Case Rep 2017;9:178–81. 10.1213/XAA.0000000000000560 28542048

[4] Apfelbaum JL Hagberg CA Caplan RA. Practice guidelines for management of the difficult airway: an updated report by the American Society of Anesthesiologists Task Force on Management of the Difficult Airway. Anesthesiology 2013;118:251–70. 23364566 10.1097/ALN.0b013e31827773b2

[5] Kim SM Kim HJ. Successful advancement of endotracheal tube with combined fiberoptic bronchoscopy and videolaryngoscopy in a patient with a huge goiter. SAGE Open Med Case Rep 2020;8:2050313X20923232. 10.1177/2050313X20923232 PMC7290263 32577281

[6] Han SH Oh AY Jung CW Park SJ Kim JH Nahm FS. The effect of the jaw-thrust manoeuvre on the ability to advance a tracheal tube over a bronchoscope during oral fibreoptic intubation. Anaesthesia 2013;68:472–7. 23573843 10.1111/anae.12176

[7] Aoyama K Takenaka I Nagaoka E Kadoya T. Jaw thrust maneuver for endotracheal intubation using a fiberoptic stylet. Anesth Analg 2000;90:1457–8. 10.1097/00000539-200006000-00044 10825343

[8] Durga V Millns J Smith J. Manoeuvres used to clear the airway during fibreoptic intubation. Br J Anaesth 2001;87:207–11. 11493491 10.1093/bja/87.2.207

[9] Asai T Shingu K. Difficulty in advancing a tracheal tube over a fibreoptic bronchoscope: incidence, causes and solutions. Br J Anaesth 2004;92:870–81. 15121723 10.1093/bja/aeh136

[10] Corda DM Riutort KT Leone AJ Qureshi MK Heckman MG Brull SJ. Effect of jaw thrust and cricoid pressure maneuvers on glottic visualization during GlideScope videolaryngoscopy. J Anesth 2012;26:362–8. 22410965 10.1007/s00540-012-1339-0

[11] Asai T Murao K Johmura S Shingu K. Effect of cricoid pressure on the ease of fibrescope-aided tracheal intubation. Anaesthesia 2002;57:909–13. 12190757 10.1046/j.1365-2044.2002.02706.x

[12] Ali MS Bakri MH Mohamed HA Shehab H Al Taher W. External laryngeal manipulation done by the laryngoscopist makes the best laryngeal view for intubation. Saudi J Anaesth 2014;8:351–4. 25191185 10.4103/1658-354X.136431 PMC4141383

[13] Levitan RM Mickler T Hollander JE. Bimanual laryngoscopy: a videographic study of external laryngeal manipulation by novice intubators. Ann Emerg Med 2002;40:30–7. 12085070 10.1067/mem.2002.125716

[14] Benumof JL. Difficult laryngoscopy: obtaining the best view. Can J Anaesth 1994;41:361–5. 8055601 10.1007/BF03009856

[15] Knopp RK. External laryngeal manipulation: a simple intervention for difficult intubations. Ann Emerg Med 2002;40:38–40. 12085071 10.1067/mem.2002.125058

[16] Ochroch EA Levitan RM. A videographic analysis of laryngeal exposure comparing the articulating laryngoscope and external laryngeal manipulation. Anesth Analg 2001;92:267–70. 11133642 10.1097/00000539-200101000-00053

[17] Adnet F Racine S Borron S. A survey of tracheal intubation difficulty in the operating room: a prospective observational study. Acta Anaesthesiol Scand 2001;45:327–32. 11207469 10.1034/j.1399-6576.2001.045003327.x

[18] Sellick B. Cricoid pressure to control regurgitation of stomach contents during induction of anaesthesia. Lancet 1961;278:404–6. 10.1016/s0140-6736(61)92485-0 13749923

[19] Roberts J Abouleish A Curlin F Patterson A. The failed intubation: maximizing successful management of the patient with a compromised or potentially compromised airway. Clinical Management of the Airway 1994;Philadelphia, PA: WB Saunders, 187–218.

[20] Johnson DM From AM Smith RB From RP Maktabi MA. Endoscopic study of mechanisms of failure of endotracheal tube advancement into the trachea during awake fiberoptic orotracheal intubation. Anesthesiology 2005;102:910–4. 15851876 10.1097/00000542-200505000-00008

[21] Ren Q Yu W Cai M. A technique to facilitate laryngeal passage during flexible bronchoscopic intubation. Can J Anaesth 2015;62:841–2. 25804432 10.1007/s12630-015-0370-7

[22] Jackson AH Orr B Yeo C Parker C Craven R Greenberg SL. Multiple sites of impingement of a tracheal tube as it is advanced over a fibreoptic bronchoscope or tracheal tube introducer in anaesthetized, paralysed patients. Anaesth Intensive Care 2006;34:444–9. 16913339 10.1177/0310057X0603400409

[23] Lee AR Yang S Shin YH. A comparison of the BURP and conventional and modified jaw thrust manoeuvres for orotracheal intubation using the Clarus Video System. Anaesthesia 2013;68:931–7. 23841798 10.1111/anae.12282

[24] Gu J Xu K Ning J Yi B Lu K. GlideScope-assisted fiberoptic bronchoscope intubation in a patient with severe rheumatoid arthritis. Acta Anaesthesiol Taiwan 2014;52:85–7. 25016513 10.1016/j.aat.2014.04.002

[25] Kumar R Sahay N Bharti B Kumar A. Laryngoscopy-assisted fiberoptic intubation in an adult with a large vallecular haemangioma. Indian J Anaesth 2020;64:907–9. 33437085 10.4103/ija.IJA_157_20 PMC7791428

[26] Garg R Nandi R Hadda V Mohan A. Conventional laryngoscopy as a rescue for fiber-optic-assisted tracheal intubation in a patient with perilaryngeal edema after rigid bronchoscopy: a case report. Lung India 2019;36:253–4. 31031350 10.4103/lungindia.lungindia_251_17 PMC6503725

[27] Kaur H Kataria AP Muthuramalingapandian M Kaur H. Airway considerations in case of a large multinodular goiter. Anesth Essays Res 2017;11:1097–100. 29284884 10.4103/aer.AER_86_16 PMC5735459

[28] Loftus PA Ow TJ Siegel B Tassler AB Smith RV Schiff BA. Risk factors for perioperative airway difficulty and evaluation of intubation approaches among patients with benign goiter. Ann Otol Rhinol Laryngol 2014;123:279–85. 24595624 10.1177/0003489414524171

[29] Gilfillan N Ball CM Myles PS Serpell J Johnson WR Paul E. A cohort and database study of airway management in patients undergoing thyroidectomy for retrosternal goitre. Anaesth Intensive Care 2014;42:700–8. 25342401 10.1177/0310057X1404200604

[30] Randolph GW Dralle H Abdullah H. Electrophysiologic recurrent laryngeal nerve monitoring during thyroid and parathyroid surgery: international standards guideline statement. Laryngoscope 2011;121: (Suppl 1): S1–6. 21181860 10.1002/lary.21119

[31] Spadaro S D’Agata M Verri M Ragazzi R Volta CA. Successful nasal intubation with a laryngeal nerve monitoring tube using bronchoscopy in a patient with plunging goiter: a case report. BMC Res Notes 2013;6:467. 24229430 10.1186/1756-0500-6-467 PMC4225601

